# Phosphatase-Coupled Universal Kinase Assay and Kinetics for First-Order-Rate Coupling Reaction

**DOI:** 10.1371/journal.pone.0023172

**Published:** 2011-08-11

**Authors:** Zhengliang L. Wu

**Affiliations:** R&D Systems Inc., Minneapolis, Minnesota, United States of America; University of South Florida College of Medicine, United States of America

## Abstract

Kinases use adenosine-5′-triphosphate (ATP) as the donor substrate and generate adenosine-5′-diphosphate (ADP) as a product. An ADP-based phosphatase-coupled kinase assay is described here. In this assay, CD39L2, a nucleotidase, is added into a kinase reaction to hydrolyze ADP to AMP and phosphate. The phosphate is subsequently detected using malachite green phosphate-detection reagents. As ADP hydrolysis by CD39L2 displays a first-order rate constant, relatively simple equations are derived to calculate the coupling rate and the lagging time of the coupling reaction, allowing one to obtain kinase kinetic parameters without the completion of the coupling reaction. ATP inhibition of CD39L2-catalyzed ADP hydrolysis is also determined for correction of the kinetic data. As examples, human glucokinase, *P. chrysogenum* APS kinase and human ERK1, kinases specific for sugar, nucleotide and protein respectively, are assayed. To assess the compatibility of the method for high-throughput assays, Z′ factors >0.5 are also obtained for the three kinases.

## Introduction

Phosphorylation is a predominant mechanism for intracellular signal transduction and enzymatic regulation. In humans, phosphorylation is carried out by more than 500 kinases [1], [2], and 30% of all proteins may be phosphorylated [3]. Through phosphorylation, extracellular signals can be relayed to the cytoplasm and cell nucleus, whereby fundamental cellular processes including the cell cycle, cell migration, cell metabolism, cell survival, as well as cell proliferation and differentiation, are controlled [4]. Due to the key regulatory roles of phosphorylation in almost every cellular activity, kinases are ideal targets for drug intervention [5], [6], [7]. However, in order to design drugs targeted to kinases, assays are required to evaluate the efficacy of drug candidates on kinase activity.

Most known kinases utilize ATP as the phosphate donor and release ADP as a by-product. Traditionally, radiolabeled ATP is used in kinase assays [8], [9], [10], [11]. Due to the high cost and strict regulation associated with radioisotope assays, numerous antibody-dependent and/or fluorescence/luminescence-based assays have been developed [12],[13],[14]. While the majority of these assays detect ATP or the phosphorylated products, only a few measure ADP accumulation.

Assays based on ADP quantification are universal, as ADP is a common product of kinase reactions. In addition, ADP-based assays are more desirable, because the rate of ADP production directly reflects the enzyme kinetics. Currently, there are two types of ADP-based kinase assays available. The first type uses coupling enzymes to convert ADP to chemicals that can be directly measured. One example is to convert ADP back to ATP through pyruvate kinase to produce pyruvate that is oxidized by pyruvate oxidase to generate hydrogen peroxide, which is further coupled to the formation of resorufin, a fluorescent molecule that can be directly detected [15]. Another example is to convert ADP back to ATP for detection using luciferase/luciferin reaction [16]. The second type is an antibody based assay, where a fluorophore conjugated anti-ADP antibody is used to detect ADP production [17], [18].

**Figure 1:**
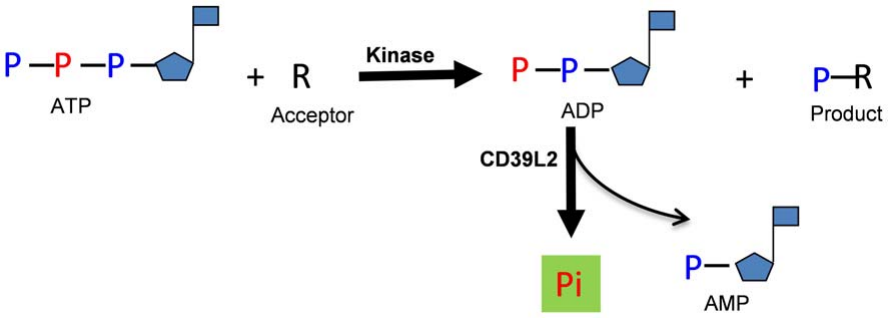
Scheme for a CD39L2-coupled kinase assay. CD39L2 selectively releases the β-phosphate of the ADP generated from a kinase reaction. The released free phosphate is detected using phosphate-detection reagents. The rate of free phosphate production reflects the kinetics of the kinase reaction.

Here, an ADP-based phosphatase-coupled kinase assay, similar to a phosphatase-coupled glycosyltransferase assay [19] is described. This assay utilizes a different nucleotidase, CD39L2 [20], to selectively release the β-phosphate from ADP (Fig.1) and the phosphate is subsequently detected using malachite green reagents. Compared to the existing methods, this assay method is simple, convenient, direct and quantitative.

## Materials and Methods

ATP, ADP, and glucose were from Sigma-Aldrich (St. Louis, MO). Recombinant mouse CD39L2 (ENTPD6), human ERK1, and the Malachite Green Phosphate Detection Kit were from R&D Systems (Minneapolis, MN). APS kinase (APSK) of *Penicillium chrysogenum* was expressed in *E.coli* (The expression clone was obtained from Dr. Andrew J. Fisher of University of California-Davis) [21]. Human glucokinase (GCK) (V16 to Q465, accession number P35557) was expressed as an N-terminal 6xHis-tagged recombinant protein in *E. coli*. Myelin basic protein kinase peptide (CVTPRTPPPSQ-OH) was custom synthesized by Proteos (Kalamazoo, MI).

A typical CD39L2-coupled kinase reaction was carried out in 50 µL of a kinase assay buffer (25 mM Tris, 150 mM NaCl, 10 mM MgCl_2_, and 10 mM CaCl_2_, pH 7.5) in a 96-well clear plate at room temperature for approximately 20 minutes. To determine the K_m_ value of a kinase, multiple reactions with varying concentrations of a substrate were performed simultaneously in the presence of fixed amounts of all other components. Each reaction was stopped with 30 µL of Malachite Reagent A and 100 µL of water. The color was developed with 30 µL of Malachite Reagent B and read at 620 nm in a plate reader (SpectraMax Plus by Molecular Device) 20 minutes afterwards. The optical density (OD) was then plotted versus the substrate concentration. For determination of the K_m_ for an acceptor substrate, if applicable, the plot was subsequently fit into the Michaelis-Menten equation using KaleidaGraph (http://www.synergy.com). For determination of the K_m_ for ATP, the plot was first corrected for the background caused by ATP hydrolysis and then adjusted with the inhibition on the coupling enzyme caused by ATP prior to fitting into the Michaelis-Menten equation. To determine the kinetic parameters of CD39L2, ADP or ATP was directly treated with the enzyme for 5 to 15 minutes and the released phosphate was detected using Malachite Reagents. When the predicted phosphate content in a reaction was above 4,000 pmol, a portion of the reaction was used for phosphate detection and the OD of the whole reaction was mathematically calculated from the measured OD. For enzymatic kinetic analysis, the substrate consumption rate for each reaction was kept less than 20%.

For determining the Z′ factors of an assay, multiple kinase reactions and same number of no-kinase negative controls were incubated in a 96-well plate and OD was measured after the addition of Malachite Reagents. The OD readings of all the reactions and the negative controls were separately analyzed for standard deviations and averages using Microsoft Excel. Z′ factors were then calculated according to the following equation [22], 
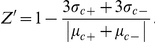


## Results

### Preference of CD39L2 for ADP

**Figure 2:**
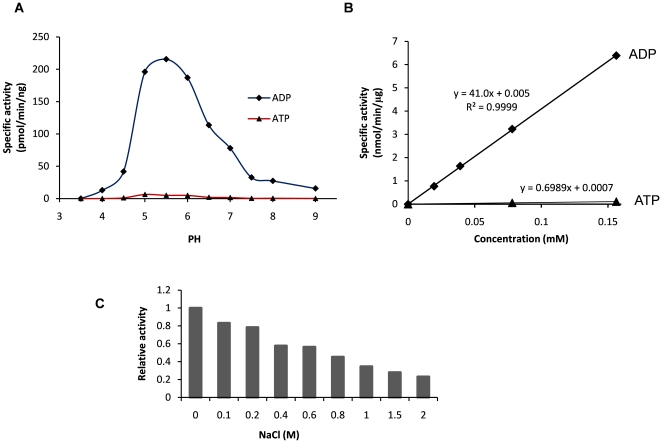
Enzymatic characterization of CD39L2. **A**) pH profile of CD39L2 activity with ADP or ATP at room temperature. CD39L2 has optimal pH at 5.5 and is selectively active on ADP throughout the pH range. **B**) First-order-rate constant of CD39L2 for ADP or ATP at pH 7.5. The specific rate constant of CD39L2 for ADP or ATP in the kinase assay buffer at room temperature was determined to be 41.0 or 0.7 nmol·min^−1^ mM^−1^·µg^−1^ (the slopes of the curves), respectively. **D**) Relative CD39L2 activity at different salt concentrations.

Several ecto-nucleotidases of the CD39/NTPDase family are active on ADP [23]. However, the nucleotidase that is able to effectively couple to a kinase reaction must also be inactive or have very little activity on ATP. Among all known nucleotidases, only CD39L2 has been reported to have such selectivity [24], [25]. The activities of this enzyme on ADP and ATP were first compared at different pH (Fig. 2A). The optimal activities were found at pH 5.5 and the preference for ADP was clear throughout the entire pH range tested. Although CD39L2 is much less active at a neutral pH, enzymatic parameters for CD39L2 were further measured at pH 7.5 (Table 1), since most known kinases are cytosolic and have optimal activity close to pH 7.5. While the K_m_ values for both ADP and ATP were around 1 mM, CD39L2 showed more than a 50-fold preference for ADP than for ATP. When ADP and ATP concentrations were significantly below the K_m_ values (<0.15 mM), CD39L2 hydrolysis of both substrates exhibited the kinetics of a first-order-rate reaction, and the specific rate constants for ADP and ATP were determined in the kinase assay buffer to be 41.0 and 0.699 nmol·min^−1^·mM^−1^·µg^−1^, respectively (Fig. 2B). CD39L2 was also found to be active in a wide range of salt concentrations, although higher levels of salt caused some inhibition (Fig. 2C).

**Table 1:** Kinetic parameters of CD39L2.

Substrate	K_m_ (mM)	k_cat_(s^-1^)	k_cat_/K_m_ (s^−1^ mM^−1^)	V_max_(nmol min^−1^ µg^−1^)
ADP	1.05	38.5	36.6	49.1
ATP	1.14	0.617	0.727	0.928

The kinetic data were measured in the buffer of 25 mM Tris, 150 mM NaCl, 10 mM MgCl_2_, and 10 mM CaCl_2_ at pH 7.5 and room temperature. For accuracy, the substrate consumption rate in all reactions was kept below 12%.

### Coupling rate and lagging time of CD39L2-coupled kinase reaction

In a coupled reaction, the efficiency for the conversion of the product to signal is critical to the success of an assay. The coupling rate (*r*) is defined as the ratio of the product that has been converted to the signal by the coupling reaction to the total product generated by the primary reaction. In a CD39L2-coupled kinase reaction, ADP is the product and the free phosphate is the signal. As the ADP concentration is likely to be maintained at low levels in the reaction, CD39L2 hydrolysis of ADP is likely to be a first-order-rate reaction. Accordingly, *r* can be calculated as the following.

The primary kinase reaction, 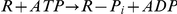
rate constant *k_1_*

The coupling reaction, 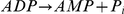
rate constant *k_2_*

The velocities of the two reactions should be,
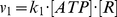
(1)

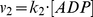
(2)


For enzyme kinetic study, because the substrate consumption should be kept as low as possible, [ATP], [R] and *v*_1_ can be approximated as a constants. Eq.2 can be considered as a degenerated form of the Michaelis-Menten equation, *v*_2_ = (*V*_max_•[*ADP*]/(*K*_M_+ [ADP]), when K_M_ ≫ [ADP]. *k*_2_ can be calculated by the determined specific rate constant, 41.0 nmol min^−1^ mM^−1^ µg^−1^ (Fig. 2B), and the amount of CD39L2, *i.e. k*_2_ *= *41.0×[E], ([E], the amount of CD39L2).

During the course of the reaction,
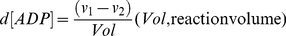
(3)


After combining Eq.2 and Eq.3, 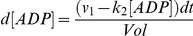 which can be rearranged to: 
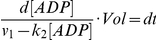


Integrating from 0 to *t*, 
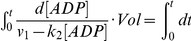


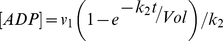
(4)


When *t* → ∞, a steady state is achieved and 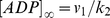
(5)


Eq. 4 can then be rewritten as,
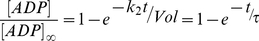
(6)


where,
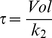
(7)


Since *Vol* and *k*_2_ are constants in a coupled reaction, τ is also a constant.

The phosphate produced (*P_i_*) by the coupling reaction can be calculated through integration of Eq.2.
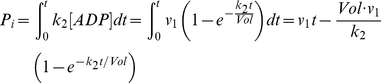



*V*_1_ *t*represents the amount of ADP produced by the kinase reaction.

The coupling rate then can be calculated as:
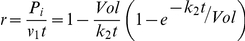



After substitution with Eq.7,
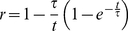
(8)


**Figure 3:**
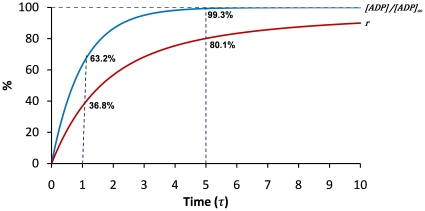
Time dependence of [ADP] and coupling rate for a CD39L2-coupled kinase reaction. *[ADP]/[ADP]_∞_* and coupling rate are plotted versus time. The time is measured as the number of the lagging time, τ. When *t* = τ, *r* = 0.368 and *[ADP]/[ADP]_∞_* = 0.632; when *t* = 5τ, *r*  = 0.801 and *[ADP]/[ADP]_∞_* = 0.993. It takes 100τ for the coupling rate to reach 99%.

Eq.6 tells how far the reaction is away from the steady state. Eq.8 shows how the coupling rate is related to time. Eq. 6 and Eq. 8 are then plotted versus time in the number of τ (Fig. 3). In particular, when *t* = τ, *r = *0.368 and [ADP]/[ADP]_∞_ = 0.632; when *t* = 5τ, *r = *0.801 and [ADP]/[ADP]_∞_ = 0.993. It is clear that τ reflects the time required to achieve steady state and therefore is called the lagging time of a coupling reaction. For any defined coupled reaction, τ can be calculated with Eq.7 and the coupling rate can be calculated with Eq. 8. In the case when τ is significantly smaller than *t*, Eq.8 can be approximated as,
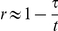


### ATP inhibition of CD39L2-catalyzed ADP hydrolysis

During the course of a kinase reaction, the ATP concentration is generally much higher than ADP, especially at the beginning of the reaction. High levels of ATP can cause two effects on a CD39L2-coupled kinase assay. First, although CD39L2 is overwhelmingly more active on ADP than on ATP, hydrolysis of ATP may become significant when the ATP concentration is high. Nonetheless, given that during kinetic assays, substrate concentrations are roughly kept constant, ATP hydrolysis caused by CD39L2 can be predicted from the enzyme kinetics and subtracted out as background. Second, ATP competes with ADP for CD39L2 activity, leading to less ADP hydrolysis and hence an underestimation of the kinase activity.

**Figure 4:**
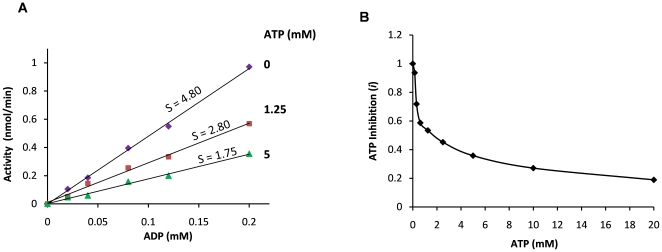
ATP inhibition of CD39L2-catalyzed ADP hydrolysis. **A**) Rate constants for CD39L2 in the presence of different concentrations of ATP. The reactions were performed with 0.12 µg of CD39L2 in 50 µL of the kinase assay buffer at room temperature. For clarity, only data at 0, 1.25 and 5 mM of ATP are shown. Slopes (s) of the curves represent the rate constants. Elevated phosphate levels in all reactions due to ATP hydrolysis were regarded as background and were subtracted out. **B**) ATP inhibition factor (*i*), the ratio of a rate constant in the presence of ATP to the rate constant in the absence of ATP, is plotted versus ATP concentration.

To assess the ATP inhibition of CD39L2 activity, rate constants of CD39L2 in the presence of different constant concentrations of ATP were determined (Fig.4A). ATP at high concentrations caused significant inhibition of the activity of CD39L2. An ATP inhibition factor (*i*) was then defined as the ratio of the rate constant in the presence of ATP to the rate constant in the absence of ATP. When *i* was plotted versus ATP concentration, a decreasing curve was obtained (Fig.4B). The value of *i* was then used to adjust the lagging time and the coupling rate by substituting *k*_2_ with *i*·*k*_2_ in Eq. 7, which became,
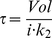
(9)


### Kinetic assay for human glucokinase (GCK)

**Figure 5:**
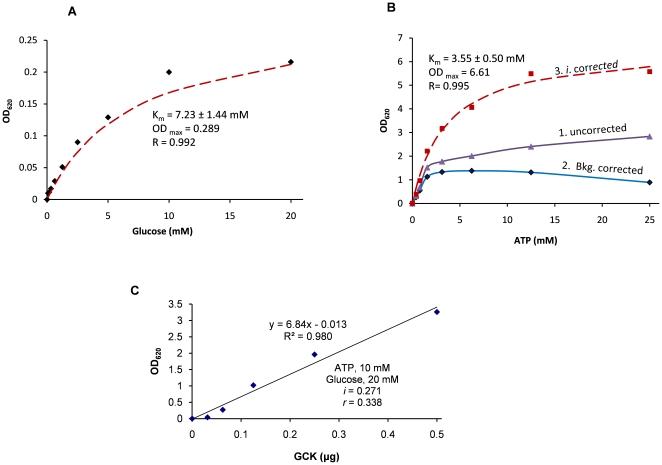
Assay for recombinant human GCK. **A**) A Glucose curve. All reactions were initiated in the presence of 0.12 mM ATP, 0.2 µg GCK and 0.3 µg CD39L2 in 150 µL assay buffer at room temperature and proceeded for 15 minutes. The reaction that contained no glucose was set as a blank and the OD readings were plotted versus glucose concentration. The curve fit the Michaelis-Menten equation well with K_m_ of 7.23±1.44 mM. **B**) An ATP curve. Reactions were performed in the presence of 20 mM glucose, 1 µg GCK and 0.1 µg CD39L2 and variable concentrations of ATP in 50 µL assay buffer at room temperature and proceeded for 25 minutes. The reaction that contained no ATP was set as a blank. For each ATP concentration, a no-kinase negative control was performed for background correction. The OD readings of the reactions (purple) were first corrected with backgrounds subtraction (blue) and further corrected using ATP inhibition factors (red) and finally fit with the Michaelis-Menten equation to obtain a K_m_ around 3.55 mM. **C**) A GCK dose curve was performed with 10 mM ATP, 20 mM of glucose and 0.2 µg CD39L2 in 50 µL assay buffer at room temperature. All reactions were proceeded for 20 minutes and the OD was plotted versus GCK input. The reaction that contained no kinase was set as a blank. The slope of the curve, 6.84 OD/µg, corresponded to a specific activity of 2416 pmol/min/µg using Eq.10 (*k_2_* = 8.2 nmol·min^−1^·mM^−1^ ; *Vol* = 50 µL; *t* = 25 min; *i* = 0.271; *r* = 0.396).

GCK catalyzes the conversion of glucose to glucose-6-phosphate and is characterized by a high K_m_ for glucose from 6 mM to 10 mM [26], [27]. GCK is expressed in insulin-secreting pancreatic β cells and hepatocytes and GCK mutations are associated with non-insulin-dependent (type 2) diabetes mellitus [28], [29]. Traditionally, the GCK assay is coupled to glucose-6-phosphate dehydrogenase to generate NADPH [27], which can be readily measured. As an example, the GCK K_m_ for glucose was first measured with CD39L2-coupled reactions. When the OD_620_ from the reactions was plotted versus the glucose concentration and fit to the Michaelis-Menten equation (Fig. 5A), the K_m_ for glucose was found to be 7.23±1.44 mM, which is well within the range of reported values.

The measurement of K_m_ for ATP requires two steps of adjustment (Fig. 5B), as ATP not only contributed to the background but also inhibited the activity of the coupling enzyme, CD39L2. To correct the background caused by ATP hydrolysis, a no-kinase negative control was performed for each ATP concentration. The background-corrected signals were further adjusted by the corresponding ATP inhibition factor *i* and finally fit with the Michaelis-Menten equation to obtain the K_m_ around 3.55 mM, which is considered to be consistent with the reported values [30], [31].

Finally, a GCK enzyme curve was performed in the presence of 10 mM ATP, 20 mM of glucose (> two fold of K_m_ for either substrate) and 0.2 µg of CD39L2 in 50 µL of kinase assay buffer at room temperature (Fig 5C). All reactions lasted for 25 minutes. Under these conditions, the coupling rate was calculated to be 0.396 using Eq.7 and Eq.8 (*k_2_* = 41 nmol min^−1^ mM^−1^·µg^−1^×0.2 µg = 8.2 nmol min^−1^ mM^−1^; *Vol* = 50 µL; *i* = 0.271 at 10 mM ATP; τ = 22.5 min; *t* = 25 min). The slope of the curve (6.84 OD/µg) was then converted to the specific activity (SA), 2416 pmol/min/µg, using Eq.10. 
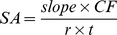
(10)


CF, phosphate conversion factor (3500 pmol/OD was used throughout this project);

*r*, coupling rate; *t*, reaction time.

### Kinetic Assay for APS kinase of *P. chrysogenum*

**Figure 6:**
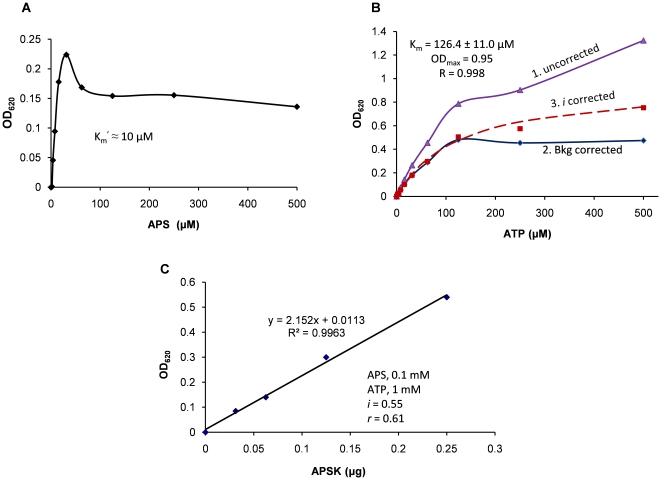
Assay for adenosine 5′-phosphosulfate kinase (APSK) from *Penicillium chrysogenum*. **A**) An APS curve. All reactions were initiated with 0.12 mM ATP, 0.1 µg of APSK and 0.3 µg CD39L2 in 150 µL assay buffer at room temperature and proceeded for 10 minutes. The OD was plotted versus the APS concentration and the apparent K_m_ (designated as K_m_′) was visually estimated to be about 10 µM. **B**) An ATP curve. All reactions were performed in the presence of 100 µM APS, 0.2 µg of APSK and 0.3 µg of CD39L2 in 150 µL assay buffer at room temperature and proceeded for 15 minutes. The obtained OD readings (purple) were first corrected by background subtraction (blue) and then corrected by the ATP inhibition factors (red) and finally fit with the Michaelis-Menten equation to obtain a K_m_ of 126 µM. **C**) An APSK enzyme curve. All reactions were performed with 1 mM ATP, 0.1 mM APS and 0.14 µg CD39L2 in 50 µL assay buffer at room temperature for 20 minutes. The OD was plotted versus APSK input. The slope of curve was converted to a specific activity, 1125 pmol/min/µg, using Eq.10 and *r* = 0.61.

APS kinase (APSK) of *Penicillium chrysogenum* is a nucleotide kinase that phosphorylates adenosine 5′-phosphosulfate (APS) at the 3′ position [32]. An APS curve was first performed (Fig. 6A). APS showed substrate inhibition above 30 µM and had a K_m_′ (substrate concentration at half maximal velocity) about 10 µM, which is consistent with the reported value [33]. An ATP curve was also performed (Fig. 6B). After the correction by ATP inhibition factor, the curve fit the Michaelis-Menten equation well. The K_m_ was then determined to be 126 µM. Finally, an APSK enzyme curve was performed with 1 mM ATP, 0.1 mM APS, 0.14 µg of CD39L2 in 50 µL of kinase assay buffer at room temperature (Fig. 6C). All reactions were stopped at 20 minutes of reaction. Under these assay conditions, the coupling rate was calculated to be 0.43 using Eq.7 and Eq.8 (*k_2_* = 41 nmol·min^−1^ ·mM^−1^·µg^−1^×0.14 µg = 5.7 nmol·min^−1^ mM^−1^; *Vol* = 50 µL; *i* = 0.55 at 1 mM ATP; τ = 15.8 min; *t* = 20 min). The slope of the curve (2.15 OD/µg) was then converted to the specific activity, 871 pmol·min^−1^·µg^−1^, using Eq.10. The difference between these determined values and the reported values [33] could be due to different assay conditions and methods, such as that the ADP was promptly removed by the coupling reaction in the current assay but might not be the case in the traditional assay.

### Specific activity determination for recombinant human ERK1

**Figure 7:**
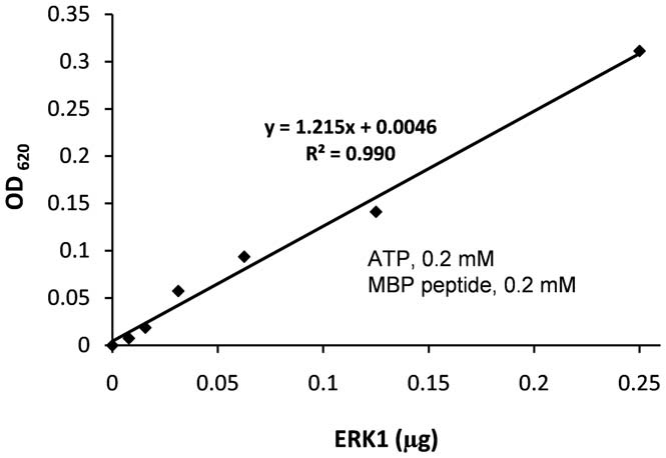
An enzyme curve for human extracellular signal-regulated kinase 1 (ERK1). All reactions were initiated with 0.2 mM of ATP, 0.2 mM of myelin basic protein peptide and 0.2 µg of CD39L2 in 50 µL assay buffer and proceeded for 15 minutes. The OD was plotted versus ERK1 input. A reaction containing all components except kinase served as a blank. The slope of the curve (1.215 OD/µg) was converted to a specific activity, 482 pmol/min/µg, using Eq.10 and *r* = 0.597.

Extracellular signal-regulated kinase 1 (ERK1), also known as mitogen-activated protein kinase 3 (MAPK3), is involved in a signaling cascade that regulates various cellular processes including proliferation, differentiation and cell cycle progression in response to extracellular signals [34], [35]. Reactions containing 0.2 mM ATP, 0.2 mM myelin basic protein peptide and variable amounts of ERK1 in 50 µL of kinase assay buffer were coupled to 0.2 µg CD39L2 for 15 minutes at room temperature and the final OD was plotted versus the ERK1 input (Fig. 7). Under these assay conditions, the coupling rate was calculated to be 0.59 using Eq.7 and Eq.8 (*k_2_* = 41 nmol·min^−1^·mM^−1^·µg^−1^ ×0.2 µg = 8.2 nmol·min^−1^ mM^−1^; *Vol* = 50 µL; *i* = 0.88 at 0.2 mM ATP; τ = 6.9 min; *t* = 15 min). The slope of the curve 1.21 OD/µg was then converted to a specific activity of 482 pmol· min^−1^ µg^−1^ using Eq.10. No comparable activity measurement for ERK1 was found in the literature; however, this result is consistent with the manufacturer's specification using a radioisotope assay.

### Z′ factor for CD39L2-coupled kinase assay

**Figure 8:**
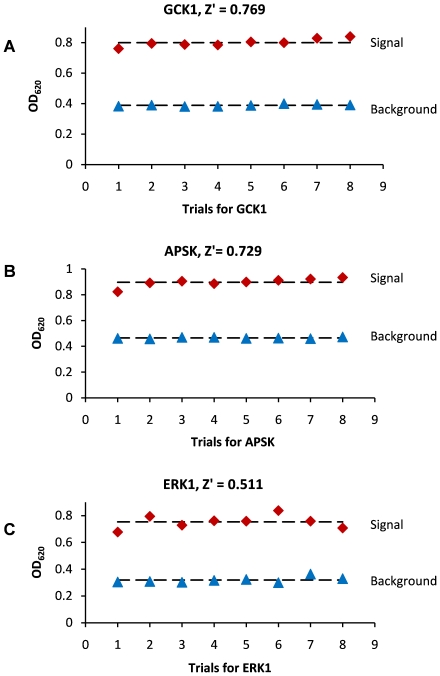
Z′ factor determination for the three representative kinases. Red diamonds represent the signal. Blue triangles represent the background. Background reactions contained all components except the kinases. **A**) Trials for GCK. Reactions were performed with 20 mM Glucose, 0.1 mM ATP, 0.64 µg GCK and 0.1 µg CD39L2 in 150 µL kinase assay buffer at room temperature for 20 minutes. **B**) Trials for APSK. Reactions were performed with 0.0625 mM APS, 0.125 mM ATP, 1 µg APSK, 0.15 µg CD39L2 in 150 µL assay buffer at room temperature for 15 minutes. **C**) Trials for ERK1. Reactions were performed with 0.05 mM MBP, 0.1 mM ATP, 0.2 µg ERK1, 0.1 µg CD39L2 in 150 µL assay buffer at room temperature for 20 minutes.

The separation between the signal and the background is essential for a high-throughput enzymatic assay. A Z′ factor quantitatively describes this separation and is used to assess the feasibility of a new method for high-throughput compatibility [22]. The CD39L2-coupled assay was also applied to the three kinases to obtain Z′ factors under the same or similar conditions as the previous activity assay (Fig. 8). In each case, a Z′>0.5 was obtained, which is considered to be excellent in high-throughput assays. To achieve better separation of the signal from the background, one can further increase the signal by increasing the amount of kinase or decrease the background by decreasing the amount of ATP.

## Discussion

For accurate kinase activity determination, coupling rate (*r*) is calculated based on the assumption that the primary kinase reaction proceeds at a constant rate and the coupling reaction proceeds at a first-order rate. Compared to second-order-rate or multiple-step coupling reactions [36], [37], single-step first-order-rate coupling reaction is much simpler in mathematics for calculating *r*. In essence, *r* is determined by three factors (Eq.7 and Eq.8): the reaction volume (*Vol*), the rate constant of the coupling reaction (*k_2_*) and the reaction time (*t*). The lagging time, τ, determined by *Vol* and *k_2_*, allows one quickly to estimate the coupling rate of a reaction. For example, it takes one τ to achieve 36.8% and 5τ to achieve 80.1% in coupling rate. Inhibition of CD39L2 by ATP results in the reduction of the rate constant and therefore the overall coupling rate. ATP inhibition is quantified as an inhibition factor (*i*). Knowing the coupling rate of a coupled reaction allows one to calculate enzyme activity without the completion of the coupling reaction.

When designing a CD39L2-coupled kinase assay, its coupling rate needs to be carefully controlled. A high coupling rate or completion of a coupled reaction is generally the goal for a coupled reaction, but it is not the case for a CD39L2-coupled kinase reaction, as a higher coupling rate is associated with a higher background caused by concomitant hydrolysis of the reactant ATP. To maximize the signal/noise ration, an ideal coupling rate for CD39L2-coupled kinase reaction may be in the range from 25 to 90%. There are several variables that may be adjusted to achieve a particular coupling rate. **First**, the amount of coupling enzyme directly contributes to the rate constant in Eq.7 and affects the overall coupling rate. **Second**, buffer conditions are not only critical for the kinase activity but also for the coupling enzyme. In this project, all assays were performed at pH 7.5 with 150 mM NaCl, which is good for many kinases but a compromise for the coupling enzyme. If an assay is performed at a different pH and salt concentration, the rate constant of the coupling enzyme should be measured under the new conditions prior to the assay to determine the amount of coupling enzyme for achieving an optimal coupling rate. Besides, Ca^2+^ or Mg^2+^ needs to be included in the new buffer, as they are the cofactors of CD39L2. **Third**, reaction time is another determinant of the coupling rate. A shorter reaction time will result in a lower coupling rate and may not be as convenient to perform. A longer reaction time will result in a higher coupling rate but may also result in excessive ATP hydrolysis and consequently higher background. In situations where a kinase activity is too low and requires a much longer reaction time, a de-coupled assay may be performed, in which CD39L2 is introduced at a later stage or after the kinase reaction is stopped, in order to minimize the ATP hydrolysis. **Fourth**, when the reaction volume is altered, the amount of coupling enzyme requires proportional adjustment to achieve the same or similar level of coupling rate. **Fifth**, although reactions at as high as 25 mM of ATP were performed, ATP concentration should be kept as low as possible to avoid unnecessary background and minimize the inhibition on the coupling enzyme. For assays that require different ATP concentrations, a no-kinase negative control is recommended for each ATP concentration to measure the background resulting from ATP hydrolysis.

The sensitivity of the assay mainly relies on the phosphate detection reagents. With 96 well plate, the lower limit for accurate phosphate detection using malachite green reagents is around 200 pmol, which corresponds to 4 µM in 50 µL solution and 1 µM in 200 µL solution. For this reason, when measuring K_m_ at lower µM range, larger reaction volume is suggested.

Kinases may also have ATPase activity, an activity that can be regarded as a transferase activity using water as the acceptor substrate. ATPase activity complicates the data analysis in CD39L2-coupled kinase assay, because it generates twice as much phosphate as the kinase activity. However, ATPase activity can be measured without the coupling phosphatase, as the γ-phosphate released from ATP can be directly detected by malachite green reagents, and no ATPase activity was detected for GCK, APSK and ERK1 in this project.

In summary, a non-radioactive phosphatase-coupled kinase assay is described here. In this assay, CD39L2, a nucleotidase, is used to couple kinase reactions to specifically release phosphate from ADP. The phosphate is subsequently detected using malachite green reagents. This assay format has several benefits. First, it can be applied to any kinase reaction that produces ADP. Second, it is high-throughput compatible because separation of product and substrate is not required and the assay can be performed in a microplate and read by a plate reader. A Z′ score above 0.5 can be readily achieved under the assay conditions. Third, the assay is more direct and quantitative because the assay measures the formation of a product and involves only a single coupling step. Finally, through converting ADP to AMP, the coupling reaction eliminates potential product inhibition caused by ADP.
